# A socio-political history of South Africa’s National Health Insurance

**DOI:** 10.1186/s12939-023-02058-3

**Published:** 2023-11-30

**Authors:** Eleanor Beth Whyle, Jill Olivier

**Affiliations:** Health Policy and Systems Division, School of Public Health, University of Cape Town, Cape Town, South Africa

**Keywords:** National Health Insurance, Universal health coverage, Health system reform, Health policy, History, South Africa

## Abstract

**Background:**

Spurred by the WHO’s endorsement of universal health coverage as a goal of all health systems, many countries are undertaking health financing reforms. The nature of these reforms, and the policy processes by which they are achieved, will depend on context-specific factors, including the history of reform efforts and the political imperatives driving reforms. South Africa’s pursuit of universal health coverage through a National Health Insurance is the latest in a nearly 100-year history of health system reform efforts shaped by social and political realities.

**Methods:**

We conducted an interdisciplinary, retrospective literature review to explore how these reform efforts have unfolded, and been shaped by the contextual realities of the moment. We began the review by identifying peer-reviewed literature on health system reform in South Africa, and iteratively expanded the search through author tracking, citation tracking and purposeful searches for material on particular events or processes referenced in the initial body of evidence. Data was extracted and organised chronologically into nine periods.

**Results:**

The analysis suggests that in South Africa politics; the power of the private sector; competing policy priorities and budgetary constraints; and ideas, values and ideologies have been particularly important in constraining, and sometimes spurring, health system reform efforts. Political transitions and pressures - including the introduction of apartheid in 1948, anti-apartheid opposition, the transition to democracy, and corruption and governance failures - have alternately created political imperatives for reform, and constrained reform efforts. In addition, the country’s political history has given rise to dominant ideas, values and ideologies that imbue health system reform with a particular social meaning. While these ideas and values increase opposition and complicate reform efforts, they also help to expose the inequities of the current system as problematic and re-emphasise the need for reform.

**Conclusion:**

Ultimately, this analysis demonstrates the context-specific nature of health system reform processes and the influence of history on what sorts of reforms are politically feasible and socially acceptable, even in the context of a global push for universal health coverage.

## Background

 Universal health coverage (UHC) is included in the United Nations’ Sustainable Development Goal 3 [1], and a number of countries are currently undertaking major health policy reforms in pursuit of UHC [2, 3]. While the definition of UHC is contested, the term generally refers to financing reforms intended to expand access to healthcare, improve quality of care and protect users from healthcare-related financial hardship [3–5]. UHC is considered to be a universal goal for health systems reform globally [6, 7], and has been described by the Director-General of the World Health Organization (WHO) as “the single most powerful concept that public health has to offer” [8].

In most contexts, UHC reforms involve extending insurance coverage to more of the population, usually by increasing the involvement of non-state and for-profit actors in healthcare provision and financing [4, 9]. However, the nature of the reforms and the process by which they are achieved will depend on a host of context-specific factors, including burden of disease, the country’s public-private mix, the capacity of the state to regulate the for-profit private sector, the power of interest groups, competing policy priorities, popular ideas about the appropriate role of the state, and political ideologies [2, 6, 10, 11]. Indeed, the pressures and constraints shaping health policy trajectories are highly variable and contextually-specific, and UHC can have a variety of ideological interpretations and be used to support vastly different social and political agendas [2, 12].

In health policy analysis paying close attention to context is considered central to understanding how and why health policy processes unfold as they do [13–15]. Because health policy processes unfold within complex adaptive health systems (themselves embedded in, and open to the influence of, their socio-political context), health policy is a product of the complex interaction of contextual factors [13, 16]. Contextual factors – including political factors (democratic norms, regime changes and political culture), the waxing and waning of ideas or ideologies (such as socialism or neoliberalism), global and national political economies and the paradigms that shape them, socio-cultural factors (like class divisions), history (such as colonialism), and what Whitehead [17] calls conjunctural considerations (accidents of timing and unexpected events) – all help to explain what happens in policy processes [13, 17–20].

Indeed, while the influence of history on health policy process is a relatively neglected topic [12], health policy and systems researchers are increasingly incorporating a historical perspective into their analyses, and a small but robust body of evidence is emerging (see for example [21–24]). This growing evidence-base demonstrates the utility of a historical perspective in deepening understanding of contemporary challenges, revealing the lingering consequences of past decisions, exposing the extent to which powerful interests groups and institutional actors are able to influence reforms over time, and indicating the boundaries for what future reforms are feasible [23–25].

In South Africa, UHC is currently being pursued through a policy proposal to implement a National Health Insurance (NHI) [26]. At present, South Africa’s health system is sharply divided between public and private sectors [26]. Both public and private sectors are governed by the Minister of Health whose mandate includes setting national policy priorities and regulating all health sector actors [27, 28]. The vast majority of the population (84%) receive means-tested (often free) care in the under-resourced and over-burdened public sector [26, 27]. The large and powerful private sector – comprised mostly of for-profit providers and not-for-profit health insurance companies known as medical schemes – serves only about 16% of the population, but accounts for just less than half of all health spending nationally [6, 27]. The private sector serves predominantly those who can afford medical scheme coverage, i.e. the socio-economic elite [26, 27]. Together, means-tested public services and voluntary health insurance protect most users from catastrophic healthcare payments [27]. However, access and quality of care received still depend in part on socio-economic status and significant inequities persist, which the NHI is intended to eliminate [26, 28]. In addition, public perceptions of poor quality care in the public sector, corruption, and general mistrust of government as a service provider exacerbate frustrations at the inequities ingrained in the health system [26, 29]. The implementation of an NHI would involve creating a single funding pool which would be used to purchase a standard package of services to ensure equitable access to healthcare for all [6, 28]. The single pool would be funded through mandatory prepayment mechanisms including taxes [6, 28].

In 2012, then-Minister of Health, Aaron Motsoaledi, used the WHO’s endorsement of UHC to defend the NHI, stating “there are people who wrongly believe that the…NHI is a pipe dream concocted by the ANC [the African National Congress]. I wish to advice [sic] them that…the World Health Organisation is actively promoting this concept and describes it as Universal Health Coverage” [30]. However, the current efforts to achieve UHC through NHI are the result of a nearly 100-year history of varied attempts to reform the health system in line with universalist principles. In addition, universalist health system reform (HSR) has been a central aim of the African National Congress (ANC) – the country’s governing political party – since it came to power in the first democratic elections in 1994 [26].

Although there is a substantial body of scholarship from Health Policy and Systems researchers, historians and political analysts on various aspects of the NHI, written at various points in the policy process^1^, no comprehensive account of the policy process in its social and political context has been published, and most contemporary scholarship on the NHI touches only briefly on the early history of HSR efforts. In this paper, we present a more comprehensive account of HSR efforts in South Africa in social and political context from 1920 to 2019. We focus primarily on national-level factors, but also consider the role of international and global factors where these are particularly helpful to explaining the NHI policy process. In doing so, we lay the foundation for further analysis of the policy process, and the technical and ideological disputes that hamper it (see Whyle 2023 [34]). In addition, by synthesising the long history of HSR efforts and the political and social contexts in which they occurred, this paper reveals how current and historical social and political realities have enabled and constrained the potential for reform, and are shaping the nature of current reform proposals.

## Methods

We conducted an interdisciplinary retrospective literature review of academic and grey literature offering insight into HSR efforts in South Africa and the global and local contextual realities in which reform processes have unfolded. The review covers the policy process beginning in the 1920s, and culminating at the end of Minister of Health Motsoaledi’s tenure in 2019.

We began the search for literature using Google Scholar to search for peer-reviewed literature on NHI, social health insurance (SHI) or HSR in South Africa. From that initial set of literature, we used a snowball approach including author tracking, citation tracking and purposeful searches for material on particular events or processes referenced in the initial body of evidence. In snowballing we also expanded the search for literature to grey literature including industry reports and briefs, policy documents, official communication, speeches, and political manifestos. We also purposefully searched for reports of surveys, relevant media articles, submissions to parliament by industry bodies and civil society, and speeches by officials in the Presidency, National Department of Health (DoH) and Treasury. The review was conducted iteratively, with the search for new material continuing throughout the process of data analysis. We continued to add literature until we felt that the information on the events, pressures and processes exerting influence on HSR efforts was sufficient to explain the observed changes in policy content and enthusiasm for reform.

Ultimately, 623 items were identified for inclusion. Nineteen items had to be excluded due to the full text being unavailable. 289 of the included items were academic texts in fields spanning African Studies, Anthropology, Development Studies, Global Health, Health Policy, Health Services Research, Medicine and Public Health, History, Economics, and Politics. 334 items of grey literature were included, including 176 media articles. The oldest item was published in 1946, although the included texts were predominantly published after 1980.

Data analysis involved reading each item and extracting relevant information into a data extraction sheet. The data extraction sheet was organised chronologically by year, and divided into socio-political context (including information relating to economic, political and social events, issues and pressures, as well as other policy processes and policy decisions happening at the time), health system context (including other health policy processes and decisions, disease outbreaks and contextual factors influencing the health system such as budget constraints), and policy process (including committees of inquiry, parliamentary hearings, and public participation opportunities).

## Results: South African health system reform in social and political context

In this paper racial categories, such as ‘black’ and ‘white’, are used to explain the history of racist social and political exclusion, and to acknowledge the continued impact of these injustices in contemporary South Africa. We recognise that racial categories have no biological or scientific basis, and that these terms are inherently problematic.

The analysis that follows presents a synthesis of the included material, organised into nine periods. The periodization emerged from the data analysis and reflects broad shifts in the trajectory of policy change, which (due to the influence of contextual factors on policy development) tend to be demarcated by socio-political junctures. Table 1 presents a summary of these periods. The first period begins in 1926, with the Pienaar Commission on Old Age Pensions and National Insurance, which signified the start of welfare policy-making in South Africa, and arguably the start of the country’s HSR efforts [35–37]. At this time, South Africa was a self-governing dominion of the British empire, having been formed in 1910 through the unification of two British colonies and two Boer (Afrikaner) republics [38–40]^2^. The 1910 Act of the Union excluded black people from political participation and spurred a series of legislative moves to formalise the racial segregation and oppression that had begun with the arrival of Dutch colonists [38, 39, 42]. An extensive system of controls was instituted to ensure black people could not compete economically with white people, and to secure the black population as a source of cheap labour for white-owned farms and mines [38, 43].

**Table 1:** Summary of the nine phases of health system reform in South Africa (Source: Author)

**1. 1926 to 1939. **The election of the Pact Government enabled the institution of a South African welfare state comprised of direct grants for the elderly and disabled (in addition to other forms of social spending). At the same time, support for HSR was growing among health professionals and government officials.
**2. 1940s and 1950s. **The election of the United Party, and the publication of the Beveridge report, combined with support of health professionals and the appointment of Gluckman as Minister of Health set the stage for HSR in the form of a centrally-funded NHS open to all South Africans. However, opposition from the Medical Association of South Africa, and the introduction of apartheid prevented its implementation.
**3. 1960s and early 1970s. **The apartheid government begins tightly regulating the private health sector. Steps included the establishment of the De Villiers Commission showing the pernicious impact of the private sector on public health services, and the introduction of the Medical Schemes Act to protect private sector users.
**4. Late 1970s and 1980s. **Increasingly organised and militant apartheid opposition, combined with pressures on the public budget forces a change in the government’s stance on private healthcare. Deregulation of the private sector allows racial discrimination to be replaced by socio-economic discrimination, while limiting political damage to the National Party. However, concern about increasing healthcare costs, fragmentation, and the abdication of the state’s responsibility to provide health services reignites calls for HSR in the late 1980s.
**5. 1990 to 1993. **As the ANC prepares to govern the ‘new’ South Africa, political and economic pressures, reflecting the hegemony of neoliberal economic ideas, push the party’s development policy toward more economically conservative proposals. In the HSR debate, these pressures, combined with the size and strength of the for-profit health sector, result in proposals that envision a continued role for private actors.
**6. 1994 to 1998. **After the first democratic election, the new government inherits significant governance and bureaucratic challenges. In the health sector these include service delivery challenges in the public sector, and cost-escalation in the private sector. The new Minister of Health initiates a series of deliberative fora for HSR, but their recommendations fail to align with the Minister’s personal values, preventing policy progress.
**7. 1999 to 2006. **The government’s refusal to roll-out an HIV treatment programme in the face of an escalating epidemic distracts policy-makers and civil society from HSR efforts, but also reaffirms the role of the state in providing health services and regulating the private sector. Recommendations for an SHI, laying the groundwork for more fundamental reforms are rejected as infeasible, and efforts to regulate the private sector to contain costs have limited success.
**8. 2007 to 2015. **Zuma is elected president of the ANC and then of the country. Zuma’s pro-poor populism is distinguished from Mbeki’s ‘cold’ neoliberalism, and Zuma reignites the HSR agenda by promising the implementation of the NHI. However, the policy development process is contentious, and Zuma’s presidency is defined by grand-scale corruption and governance failures that undermine public trust in the state.
**9. 2016 to 2019. **In the shadow of state capture, Motsoaledi continues to drive the NHI policy process forward, hampered by contention surrounding the role of the private sector. Ultimately, Motsoaledi’s stance on private sector involvement softens, but concern about the capacity of the state to regulate the private sector, deliver public health services, and manage NHI funds persists.

### 1926 to 1939: The emergence of the welfare state and growing support for universalist health system reform

South Africa’s journey towards HSR begins in this context. The coalition between English capitalists and Afrikaner farmers that enabled unification also established English control of South Africa’s economy that would prove remarkably persistent [39]. However, by 1924, there was growing discontent with the leadership of the ruling party – the National Party – which was seen to be promoting the interests of capital above those of ‘ordinary’ white South Africans [44]. The ‘Pact Government’ – a coalition between the socialist English Labour Party and the nationalist Afrikaans National Party – won power in the 1924 election and began instituting policies that combined a welfare state with a racialised labour market to protect white workers from competition from black workers, and from the hardships resulting from modernisation and urbanisation [39, 44, 45].

As part of this project, in 1926, the Pienaar Commission was appointed, which laid the foundation for the 1928 Old Age Pensions Act [35–37], and was the first public commission on healthcare to reference National Health Insurance [46]. However, the recommendations of the Pienaar Commission prompted a conservative backlash and were not immediately implemented [35, 47].

In 1929, under a National Party government no longer influenced by the concerns of the Labour Party (which had split from the National party in 1928 and performed disastrously in the 1929 elections), a new commission was established – the Carnegie Poor White Commission [35, 37]. The ‘reactionary’ Carnegie Commission argued against welfare programmes that ‘put cash in the pockets of the poor’ on the grounds that they gave the impression such ‘charity’ was a right and the duty of the state, and that the Commission’s recommendations were antithetical to the building of a welfare state [35, 37]. The central tension between the Pienaar Commission and the Carnegie Commission was that the former offered a structural analysis that saw the alleviation of poverty as the responsibility of the state, whereas the latter offered an individual analysis focusing on ‘psychological traits’ as the cause of poverty, and suggested ‘self-reliance’, ‘self-help’ and ‘poor relief’ run by the church as the solution [37].

Despite the backlash and the recommendations of the Carnegie Commission, the event of the Great Depression and South Africa’s consequent recession made a strong argument for the welfare state, and the gold-fuelled growth of the 1930s staved off the worst effects of the global economic depression and imbued the state with financial means and capacity to implement the recommendations of the Pienaar Commission at scale [37, 47, 48]. By the end of the 1930s, South Africa had a well-developed institutional framework for social welfare (albeit restricted to white and coloured people, and excluding black and Indian people despite their contribution to general tax revenue), including major spending on old-age and disability pensions [35, 49].

The 1930s also saw a gradual but steady increase in calls for universal HSR. In 1931, the president of the Medical Association of South Africa (MASA), Francis Napier, penned a plea, published in the South African Medical Journal, for a state-run ‘unitary medical service’ that would allow for better coordination of preventive and curative services, and would ‘meet the needs of the whole population’ [36]. The editors of the journal dismissed the idea, but also acknowledged that it reflected the beliefs of a growing number of professionals [36]. In 1935, the idea of a ‘sate medical service’ ‘without distinction of race or colour’ was proposed in the House of Assembly [36]. The proposal was rejected on financial grounds, but reignited debate on the topic and found a more receptive audience among health professionals seeking security after the economic shock of the Depression [36, 48]. A Committee of Inquiry into NHI was established by the Public Health Department in 1935, which recommended, conservatively, an NHI to cover urban employees of all races earning below a certain threshold, and in 1939 prominent public health doctor and United Party member of parliament, Dr Henry Gluckman, voiced support for an NHI [36, 48, 50]. While the calls were once again dismissed as impractical, by 1940 those in favour of establishing a national health service (NHS) made up an influential lobby including many of the country’s most eminent physicians [36, 48, 50]. Thus, by the end of the 1930s, alongside a rapidly expanding social welfare programme resulting from the Pienaar Commission, there was also growing support for HSR. Together with the support of medical professionals and political leaders, the electoral victory of the United Party in 1938 set the stage for revolutionary reform of the health system in the 1940s.

### 1940s & 1950s: Health system reform is nearly achieved, but prevented by the introduction of apartheid

The establishment of South Africa’s first primary health centres (beginning in 1940) by Dr Sidney and Emily Kark demonstrated impressive results with well-kept statistics, and Treasury quickly made funds available to support the development of more health centres [36, 51, 52]. Simultaneously, MASA (or a group of radical doctors within MASA) began advocating for the establishment of an NHS funded through general taxation, including a 1941 pamphlet using the language of ‘socialised medicine’ and calling for the elimination of competition and commercial elements from health care [36, 48, 53].

Britain’s landmark Beveridge report – which described a ‘radical,’ ‘utopian,’ and ‘visionary’ plan for the introduction of a universal^3^ welfare state funded through general taxation – was released in 1942 to euphoric popular reception, and prompted global interest in welfare state-building, including in South Africa [40, 55, 56]. Gluckman was appointed as chair of the official National Health Services Commission, which released a detailed report on the potential for a state-run health service in South African in 1944 [53]. The Gluckman report drew inspiration from the Beveridge Report, and recommended the implementation of a centrally controlled NHS, funded through a national health tax that would deliver healthcare free to all South Africans regardless of any criteria other than need, including race or socio-economic status [7, 36, 56]. Gluckman’s recommendations focused on primary and preventive healthcare, provided at local, racially-segregated health centres (like those established by the Karks), and a diminished role for the private sector (which had been allowed to grow significantly in the preceding years), arguing that doctors should be state employees^4^ [36, 51, 58, 59]. Both the Beveridge report and the Gluckman report displayed a revolutionary zeal and an appetite for bold, transformative change. The Gluckman report acknowledged that the proposals “may, perhaps be described as revolutionary for those who look to tradition and precedent as their guide” while the Beveridge report stated that “a revolutionary moment in the world’s history is a time for revolutions, not for patching” [60].^5^ Thus, when Gluckman was appointed Minister of Health in 1945 [56, 61] health system transformation may well have seemed, if not inevitable, imminently possible.

However, unlike in Britain, a host of factors conspired to prevent health system transformation in South Africa. Firstly, MASA’s support of the NHS was conditional on the provision that doctors be allowed to continue in private practice, that curative care through health centres would be restricted to the very poor, and that the reforms did not include free hospital services, which ultimately resulted in MASA opposing the implementation of an NHS [36, 56, 61]. MASA’s opposition to the proposals was in part a function of existing vested interests in private provision, and in part a consequence of an ideological regression and growing distaste for ‘socialist tendencies’ [36, 56].

Secondly, at this time – as urbanisation, landlessness and unemployment made the issue of poverty among black people more readily apparent – the United Party government moved to deracialise the welfare system by extending welfare benefits to black people (albeit not at the same rates as for white and coloured people) and relaxing restrictions on spending on schools for black children [35, 47, 62]. This de-racialisation of the welfare system led to a severe backlash from large segments of the white population and contributed to the defeat of the United Party at the hands of the National Party in the 1948 election (despite the United Party back-tracking on the policy prior to the election). The National Party’s electoral campaign included the promise to implement apartheid – an oppressive system of institutionalised racist segregation and discrimination against black, coloured and Indian South Africans, alongside a state-directed programme of economic and social protections for ‘less well-off’ white South Africans (largely Afrikaners) [26, 38, 44, 45, 59].

Under the National Party, there was little government support for the continuation of the health centres. With the poor black majority unjustly disenfranchised, those who stood to gain the most from the implementation of the NHS lacked political power to vote for it [61]. The death of Gluckman’s successor, Minister Stals, intensified government opposition to the health centre concept [36, 56]. Most health centres were closed or converted to curative, outpatient departments [36].

In this context the ANC – an organisation, established in 1912 to oppose the political oppression of the black majority, which would become the cornerstone of the anti-apartheid movement, and democratic South Africa’s ruling party [41] – took up the mantle of universalist health system reform. Likely influenced by the thwarted promise of the NHS and the health centres, the ANC’s landmark Freedom Charter, published in 1955, called for a state-run preventive health scheme, and universal free medical care and hospitalisation for all [63–65]. The Freedom Charter also called for the redistribution of land and mineral wealth [63].

The period between 1940 and 1960 encompassed the rise and fall of South Africa’s first attempt at HSR, with the introduction of apartheid scuppering efforts to implement an NHS. The idea for HSR presented in the MASA pamphlet, Beveridge Report, the Gluckman Commission Report, and the ANC’s Freedom Charter would continue to influence HSR efforts in South Africa, but another opportunity for radical reform would not arise before 1994.

### 1960s and early 1970s: the Apartheid government increases regulation of private healthcare

While an NHS was no longer on the policy agenda, in the 1960 and 1970s the apartheid government’s response to the growing for-profit private health sector did lay the foundations for a regulatory state that protected its citizens from market forces, albeit in a context where most South Africans were not recognised as citizens. By 1960, 80% of white South Africans had private health insurance [66]. Recognising the need to protect the users of the private health sector from the consequences of inadequate coverage, the apartheid state began to regulate medical schemes [66]. The Medical Schemes Act (MSA) 72 of 1967 introduced minimum benefits, eliminated risk-rating, and set reimbursement rates so that scheme members could be sure the fee charged by doctors would match what their scheme reimbursed [66].

This move aligned with the general attitude of the National Party towards the private health sector that persisted until the late 1970s – the government understood the provision of health services to be a responsibility of the state (in line with the global primacy of welfarist views in the post-war era), increasingly took over control of the not-for-profit mission hospitals providing care in rural areas, and tolerated but tightly regulated the for-profit private health sector was [53, 67]. The 1974 De Villiers Commission into Private Hospitals and Unattached Operating Theatres argued that human resource drain from the public to the private sector (as a result of higher wages in the latter) was contributing to vacant public sector posts and undermining the strength of the public sector [53, 67]. The Commission further argued that the state not only had a responsibility to ensure adequate standard of care in all sectors, but also that the state should act as provider of hospital services as far as possible, and ultimately resulted in stricter regulation of private hospitals [53, 68].

Thus, in this period the role of the state in healthcare, with respect to regulation of non-state health services and the responsibility of the state to provide healthcare services, was reaffirmed. However, despite being more tightly regulated, the private health sector continued to grow [7, 53], and subsequent decades would cement the position of for-profit healthcare as a major actor in future HSR efforts.

It is also the case that, in this period, the health system served as a tool for surveillance and enforcement of apartheid policies. Many South African health professionals were complicit in the systematic denial of the right to health for black South Africans and either actively supported or passively allowed these human rights abuses, including state-sanctioned violence and torture [66, 69, 70]. At times, health professionals actively participated in the apartheid state’s security apparatus, for example acting as expert witnesses and giving testimony in the interest of the security forces, and used their elevated social standing to defend apartheid health policies [70]. The South African Medical Journal, for example, published articles defending apartheid and suppressed articles that criticized apartheid policies [70]. Perhaps the most egregious and infamous example of health professionals acting as operators of the apartheid government was the involvement of state-employed doctors in the death of renowned anti-apartheid activist Steve Biko. Biko died in 1997 after being beaten and sustaining brain damage during ‘interrogation’ by security police, being left lying on the floor of a jail naked and manacled for several days, and being transported over 1 000 kms manacled on the floor of a Landrover [69]. The doctors who consulted Biko in the five days before his death (including the district surgeon, chief district surgeon, and a private specialist physician) concluded that he was ‘malingering’ or ‘shamming’ despite clear evidence of extensive brain-damage, and failed to recommend either improvement in his conditions or medical intervention [69].

### Late 1970s and 1980s: Neoliberalism, financial crisis, and political pressures force privatisation and deregulation of private healthcare

In the late 1970s and 1980s, a combination of political forces (in the form of growing opposition to apartheid), economic concerns, and global ideological influences began to force a shift in the National Party’s relationship to the private sector. Firstly, the 1970s saw increased, more militant, opposition to apartheid from the black majority, the emergence of a lively anti-apartheid civil society, and a strengthening of black trade unions [71, 72]. In addition, the institution of a new Constitution in 1984 inadvertently fuelled aspirations for political and economic power among the black majority [71, 72]. In response, the National Party began to make certain concessions with the aim of gaining the cooperation of a segment of the black population, including reducing racial discrimination in old-age pension and other grant programmes, extending rights to home ownership, and reforming some discriminatory labour laws [49, 71]. At the same time, South Africa’s economic difficulties were exacerbated by pressure from anti-apartheid advocates on local and multi-national corporations to restrict capital flows in and into South Africa [57, 73]. In addition, pressure on the public budget was increasing due to a growing budget deficit; increasing resources needed in defence, security and policing to maintain political stability in the face of anti-apartheid activism 6; and increases in the cost of public health provisions due to technological development, an aging population and rapid urbanisation [53, 72, 74, 75]. In the face of these budgetary pressures, de-regulating the private sector to enable black people to enjoy private sector services was more feasible than extending welfare services to black people [53].

Secondly, around this time neoliberal economic policies were gaining popularity among industrialised states, and were increasingly being prescribed as a solution to the challenges of developing countries trying to recover from the debt crisis of 1985 [53, 76]. Neoliberal economic policies included fiscal discipline, limited public expenditure, deregulation of the private sector, privatisation and trade liberalisation [76].

In the health sector, the government’s response to budgetary pressures reveals an acceptance of, and commitment to, neoliberal economic ideologies that legitimate the state’s abdication of the responsibility for the provision of healthcare, the privatisation of service delivery and a focus on individual responsibility [73, 75, 77]. In 1986, the Browne Commission of Inquiry into Health Services, clearly influenced by a commitment to privatisation, conceded that there was no evidence that the private sector is more efficient, and that privatisation had no benefit to users, but nonetheless supported deregulation of medical schemes, including the acceptance of risk rating, threshold payments, and co-payments and deductibles [75, 78, 79]. In “an ironic reversal of the Gluckman commission” the Browne Commission also said that the development of primary health centres should be determined only *after* accounting for the likely expansion of the private sector [53].

A series of neoliberal policies were enacted in this period. Licensing requirements were relaxed to encourage construction of private hospitals [73]. Public sector fees for paying patients were rapidly increased, to the point that it was more expensive for ‘middle class’ patients to obtain out-patient services in the public sector than to visit a private provider [73, 80], and medical scheme membership was opened to people of all races (having been restricted to white people heretofore) [7, 78]. The Medical Schemes Amendment Act – pushed through in the dying days of apartheid – abolished guaranteed payments to providers, removed mandatory minimum benefits, re-enabled risk rating, and excluded many of the most vulnerable from medical scheme coverage [31, 57].

De-regulating and de-racialising the private sector, rather than actively extending public welfare services to the black population, also had important political benefits. Firstly, the policies appealed to the interests of the existing English business class and emerging Afrikaner entrepreneur class [73, 75, 81]. Secondly, by transferring responsibility for the provision of healthcare onto the private sector, the state could dampen political tensions while ensuring that the racial hierarchy was sustained by wealth disparities between racial groups, replacing explicit racial discrimination with economic discrimination [75, 82]. Thirdly, the move served to undercut apartheid opposition, because it enabled urban black, coloured and Asian workers with medical scheme coverage (a population growing as a result of trade unionisation) to access high quality private sector care, while those in rural areas continued to rely on the inadequate public sector [53, 72, 80].

The result of these policy decisions was a dramatic expansion of the private health sector, particularly for-profit hospitals,^7^ driven largely by increasing medical scheme membership among black people [73, 78]. However, as the private health sector grew, the challenges associated with private provision of healthcare became increasingly clear. Medical scheme membership fees rose dramatically over the 1980s, far outstripping inflation [73, 83]. The re-introduction of risk-rating undermined cross-subsidisation and solidarity within schemes [74, 84], and private sector costs rose rapidly as a result of fee-for-service payment mechanisms, and supplier-induced demand [80, 85].

In addition, the growth of the private sector exacerbated the already extreme fragmentation of the health sector. By the early 1980s the distinct health authorities operating at different levels of the country included provincial authorities, municipal authorities, the Central Department of Health and Welfare, and ten separate Ministries of Health for each of the ‘bantustans’^8^ responsible for all health services in the territory [71, 72, 87]. This is in addition to the private sector, which included a rapidly expanding group of for-profit providers, private medical schemes, industry heath providers (such as hospitals owned by mining companies), and non-profit organisations [87]. The 1983 Constitution added three more departments of health, as ‘own affairs’ departments were created for white, coloured and Indian populations [58, 72, 87].

In this context, in the latter part of the 1980s, the debate on NHS gained renewed attention among progressive civil society actors and academics who were concerned about the state’s abdication of responsibility for healthcare provision, fragmentation, and rising inequities in access and quality of care between public and private sectors (and therefore between the rich and poor) [31, 80].^9^ This attention, and the slew of research and strategy proposals it produced, informed the policy ideas of the ANC, which by the early 1990s was preparing to lead the country into the new democracy [31].

### 1990 to 1993: the ANC’s policy proposals are constrained by political and ideological pressure

However, the ANC’s internal policy debates were not immune to the influence of increasingly powerful neoliberal ideas, and between 1990 and 1994 the ANC faced considerable pressure to moderate their policy proposals. Historically, the ANC had been closely affiliated with socialist organisations including the Soviet Union and the South African Communist Party (SACP), with which the ANC had a long-standing alliance that was foundational to the fight against apartheid [91, 92]. Much of the ANC’s early development policy, including the Freedom Charter, had socialist overtones, and by the late 1980s SACP members dominated the ANC leadership [64, 92, 93]. In addition, the alliance between the ANC, the SACP and the Congress of South African Trade Unions (COSATU) – which operated as the dominant and most progressive arm of South Africa’s budding trade-union movement – was key to the ANC’s prospects of electoral victory, and allowed COSATU to ensure that the interests of the working class were reflected in ANC policy [94].

In the early 1990s, however, a significant tension emerged between the ANC’s traditional socialist rhetoric, and the need to avoid alienating key allies, including the emerging black capitalist class [95, 96]. For example, on the day of his release after 27 years of imprisonment, in February 1990, Nelson Mandela reaffirmed the radical redistributive principles, including nationalisation, that had come to signify liberation to many of the ANC’s supporters [95]. Mandela’s statement created fears of ‘expropriation without compensation’, and had a negative impact on the stock market, and the ANC faced significant pressure to soften its stance [93, 95, 97]. Similarly, the 1990 ANC Discussion Document on Economic Policy reflected the influence of COSATU and called for nationalisation of recently privatised public utilities and mining, increased taxes on corporations and rich, and redistributive economic policies [95]. Within the party, the discussion document sparked debate between moderate economic thinkers keen to encourage growth by appealing to international investors as ‘business-friendly’, those who felt that the abandonment of radical socialist rhetoric constituted a betrayal of the party’s grass-roots supporters, and trade-unionists who were wary of kowtowing to big business [76, 95]. Similarly, the 1994 Reconstruction and Development Plan (RDP), which would form the basis of the ANC’s economic policy until 1996, was drawn up by a former COSATU member, in response to a COSATU ultimatum, and was relatively pro-poor, deprioritised investor confidence and emphasised the state’s obligation improving social welfare through decommodification, rural development and affirmative action [95, 98]. However, the RDP as implemented was significantly more conservative and neoliberal (discussed further below) [31, 83, 99].

Ultimately, two dichotomous economic and political positions emerged: The liberal argument was that privatisation and free market principles would not only spur economic growth, but were also an appropriate redress to apartheid given that apartheid was a drag on growth and that black people would thrive given economic opportunities [73, 76, 95]. The radical argument was that apartheid and capitalism were ideologically intertwined, that South African business was complicit in apartheid, and that nationalisation and radical redistribution were necessary to liberation [73, 95, 100].

Prior to 1994, the liberal side of the debate gained power as the ANC sought to alleviate ‘white fears’ and boost business and investor confidence, and ANC policies shifted away from radical redistribution, and towards fiscal discipline [76, 95, 101]. This shift can be understood in part as consequence of the precarious fiscal position of the country at the time (by 1993 the budget deficit was nearly 8% of GDP and the possibility of a debt trap loomed) [47, 102]. However, it can also be interpreted as an inevitable consequence of the hegemony of neoliberal ideas, and, in part, as a result of systematic efforts on the part of international business elites to ‘educate’ and ‘persuade’ ANC leaders to adopt pro-market policies [97, 100, 103]. The World Bank recruited ANC officials to work in Washington in the early 1990s, and select ANC leaders underwent training at Goldman Sachs in New York, while other ANC officials were recruited to work with the World Bank in Washington, which Cronin argues was a clear attempt by global capital to create a cadre of neoliberalists within the ANC [96, 103]. In addition, the Consultative Business Forum – established in 1988 as a progressive forum to allow business to contribute to the promotion of a ‘fair and just society’ in a ‘non-racial democracy,’ and a ‘successful economy’ – facilitated key meetings between the National Party and the ANC, and strengthened the relationship between the ANC and South African business [100].

In 1991, in a speech in the USA, Mandela stated “the private sector must and will play the central and decisive role in the struggle to achieve many of [the ANC’s] objectives…The rates of economic growth we seek cannot be achieved without important inflows of foreign capital” (quoted in [31]). Similarly, the ANC’s 1992 Draft Policy Guidelines were appreciably more ‘business-friendly’ and framed the private sector as a ‘dynamic partner’ [95, 97].^10^

In the HSR debate, there was a corresponding acceptance of the role of the private sector. The debate became defined by two opposing schools of thought. The first, recognising the rapid cost spiral in the private sector and concerned about increasing inequalities between public and private sectors, argued for the establishment of a single NHI, in which mandatory contributions by employees would be combined with general tax revenue to buy healthcare from a mix of public and private providers [73]. The second, influenced by neoliberal principles and concerned that the strength of the private health sector made the first option infeasible, suggested leaving the private sector to continue to service those who could afford it, and concentrating on improving the public sector for the provision of adequate care to the poor [73]. Across both sides of the debate, there was growing acceptance of the ‘infeasibility’ of a purely public tax-funded NHS, given the size and (political) strength of the private sector [73]. The ANC’s 1991 discussion document – ‘Towards Developing a Health Policy’ – suggested a tax-funded, unitary NHS in which most provision would be public, but that would allow for the continued existence of private health care [104, 105]. More generally, reform debates began to prioritise reducing the funding gap between public and private sectors over unifying the health system, and focused on establishing the appropriate level of private sector involvement [31, 73, 106]. However, even after the first democratic elections, the tension between radical socialism and economic conservatism would shape of the ANC’s development agenda, and hinder HSR efforts. While the change of government presented an opportunity for progress, a failure to reach consensus would prevent HSR in the 1994 to 1998 period.

### 1994 to 1998: Possibility for health system reform is limited by ideological differences and neo-liberal macro-economic policy

In addition to this ideological tension, the new government also faced significant economic, bureaucratic and governance challenges. The interim constitution held that, to facilitate the democratic transition, all parties winning at least 10% of the vote would form a coalition government called the Government of National Unity (GNU). The ANC won 63% of the vote, and two-thirds of the seats in parliament, and Mandela was appointed president of the country and leader of the GNU [31, 101]. However, the civil service was bloated, inefficient, and corrupt, with state-private sector relations that enabled rent-seeking and patronage, and the negotiated transition had guaranteed existing civil servants their positions for five years, preventing a radical overhaul of the civil service [31, 76, 107]. Furthermore, the transition shifted liberation leaders inexperienced in governance into powerful positions, and the appointment of many activists to government positions undermined the country’s previously flourishing civil society and muted critical engagement on policy [31]. In addition, the ANC inherited a failing economy, with gross national product growing an average of only 8% per annum over the previous decade, and the transition took place in a global environment in which the mobility of capital severely constrained the ability of states to regulate and control it [57, 76].

Parallel challenges affected the health sector. Quality of care in public sector hospitals had been steadily declining since the mid-80s, equipment shortages were rife, and a shortage of human resources in the public sector 11 was being exacerbated by the ‘brain-drain’ to the private sector [73]. Under the previous administration’s privatisation policy, the public sector had also been subject to sudden budget cuts and dramatic decreases in public expenditure [73]. In addition, the health system was hospi-centric (in 1995, 76% of public health expenditure went to hospitals) with limited investment in primary and preventive services [57, 108]. Despite these challenges, the ANC was committed to drastically improving access to health and welfare services. Within the first 100 days of the Mandela presidency the Free Care policy was announced, making all healthcare free for pregnant women and children under six (albeit with no corresponding increase in funding to accommodate the dramatic increase in uptake) [31, 109].

In the private sector, cost-containment and accessibility were the major challenges. Medical scheme membership rates were increasingly unaffordable, and between 1993 and 1994, 200 000 people lost medical scheme coverage [83]. Following the rapid growth of the for-profit hospital sector in the late 1980s, by the early 1990s it was clear that consolidation of control, market failures and perverse incentives were undermining the stability of the sector as a whole [57]. Fee-for-service reimbursement mechanisms were creating supply-induced demand and driving up costs, and medical scheme contribution rates were consistently increasing, and were not matched by increasing benefits [57]. By the early 1990s, three hospital groups owned almost 45% of all private sector beds, and some doctors had vested interests in for-profit hospitals, resulting in unnecessary hospitalisations and further driving up costs [57]. At the time, private sector users, who until 1995 were not permitted to choose to receive care in the public sector, were at the mercy of the market [57]. Many schemes refused to enrol members over the age of 55, and either refused coverage to HIV-positive people or offered them drastically reduced benefit options [66]. Nonetheless, the rapprochement between the state and the private health sector held – in part, as a result of the acceptance of neoliberal ideas about the appropriate role of the state as regulator in a system of private sector delivery [31].

In this context, the ANC began the policy process to transform the health system. Immediately after the election, in May 1994, the ANC released a discussion document – *A National Health Plan for South Africa* – outlining the plans for health sector reform [110]. The document stated that “a single comprehensive, equitable and integrated NHS will be created and legislated for” but was carefully worded to avoid alienating important stakeholders, and called for a process of consultation with “all interested parties, including employer, labour, professional, medical aid, and health insurance organisations” and for “detailed planning for implementation of an NHI if there is sufficient consensus on this option” [110]. The new Minister of Health - Nkosazana Dlamini-Zuma, a medical doctor with a fairly radical stance on redistribution and redress of inequalities [31, 111] – established the Directorate of Health Financing and Economics to coordinate policy development for health financing reform [31]. In June 1994, a Health Care Finance Committee (HCFC) was established to “examine appropriateness and feasibility of establishing an NHI system, or for other models to enable all South Africans to have access to comprehensive health services at an affordable cost” – the first of a series of special structures for the development of healthcare financing policy [31]. The committee included an influential Australian economist, Dr John Deeble, who advocated for radical reforms in the form of a universal NHI under which private health practitioners would be nationalised [31, 112]. While most members of the HCFC considered the Deeble model politically and financially infeasible, Deeble’s ideas aligned with the Minister’s personal values [31, 112]. The report outlined three possible options for health financing reform distinguished by coverage (universal or restricted to contributors) and benefits (primary healthcare (PHC) only or PHC and hospital care), but ultimately recommended the most moderate option – an SHI ensuring comprehensive benefits for contributors and their dependents, with existing medical schemes acting as financial intermediaries – as the only politically feasible option [31, 113].

However, when the report was leaked to the press, it received significant public attention.^12^ The media described the NHI as ‘socialist,’ ‘sinister’ and a ‘threat’, and perpetuated a narrative that Dlamini-Zuma had a ‘hidden agenda,’ was ‘gagging’ those who would speak out against the plan, and was ‘pushing through’ the NHI, despite the HCFC having rejected the plan, by simply appointing a new committee (see for example [114–120]).^13^

Likely because the HCFC’s recommendation did not align with the Minister’s preferences, the committee's recommendations were not taken up [31, 112]. Instead, another committee was established – the Committee of Inquiry into NHI, co-chaired by Minister’s aide Olive Shisana [112, 121]. While there had been no process of wider consultation in the HCFC, the Committee of Inquiry undertook wide-ranging consultation [31]. The terms of reference for the committee had initially been restricted to planning for the introduction of an NHI, but one of the chairs threatened to resign if they were not broadened to include consideration of other insurance-based models [31, 112]. The Committee of Inquiry also included Treasury officials who argued that funding the NHI through general taxation was not in line with the country’s broader macro-economic policy – the RDP [31]. The committee relitigated the Deeble option, and concluded, once again, that it was not feasible – ultimately including in the report alternative, more palatable SHI models (alongside recommendations for strengthening the public health system) that would incite less backlash from the private sector [31, 112]. The recommended SHI model would cover hospital services for contributors only (because PHC was now free in the public sector), with reimbursement rates restricted to the cost of care in the public sector [31, 32]. However these recommendations were opposed by Treasury and received little wider attention [31, 112].

The Committee of Inquiry also called for a technical committee to take these recommendations forward – leading to the establishment in 1997 of the SHI Working Group and the Medical Schemes Working Group [31]. The SHI Working Group – a small and low-profile group of six analysts and DoH officials – was tasked with developing a detailed proposal for an SHI for public hospitals [31, 112]. The working group was also asked by Minister Dlamini-Zuma to reconsider the Deeble option, but once again rejected the idea as financially infeasible [31]. Instead, the Working Group recommended an SHI scheme targeted at employees who opted against (or could not afford) private health insurance, and made no effort to ensure cross-subsidisation between income groups – indicating a shift away from equity-oriented reforms and a concession to Treasury’s concern that the high-income earners not be ‘over-taxed’ for reasons of ‘fairness’ [31, 32, 106].

Minister Dlamini-Zuma’s ideological commitments aside, the Deeble option was also out of step with the pressure for health policy to conform to broader neoliberal trends [99]. The influence of the World Bank and remaining conservative bureaucrats (some of whom were apartheid-era civil servants) meant that the radical left-leaning elements of the RDP were dimmed in the white Paper on Reconstruction and Development released in November 1994, which shifted towards a contraction of the public sector, protection of property rights, and exposure of manufacturing to international competition [31, 83]. In 1996 the leftist-influenced RDP was replaced by GEAR – a purely neoliberal macro-economic policy influenced by the International Monetary Fund and the World Bank that emphasised free-market capitalism, fiscal conservatism, privatization and tax reductions for the rich [64, 109]. GEAR “codified liberalization as the official ideology” of the government [103]. In the health sector, GEAR’s effect was to constrain health care spending, undermine health system transformation processes, and discourage regulation of the private sector [64].

Stagnating financing, in combination with the explosion of HIV/AIDS and a series of related political scandals embroiling the DoH, largely pushed NHI off the agenda beginning in the mid-90s. By 1995, HIV prevalence among ante-natal clinic attendees was 4.3%, and the epidemic was spreading rapidly [57, 121]. In February 1997, a Cabinet press release announced the development of a new AIDS treatment, known as Virodene, by local researchers, Ziggie and Olga Visser [107, 122, 123]. After hearing claims by the researchers that pharmaceutical companies with vested interests were blocking research because it threatened their profits, as well as what Mbeki (then deputy president) described as ‘moving’ testimonies from AIDS patients being treated as part of an ‘unofficial’ clinical trial, Cabinet resolved to help the researchers gain approval for formal clinical trials [123, 124]. However, the biomedical community, drug regulatory authority and Medicines Control Council (MCC) found that the drug had not passed basic biological or animal experimentation and had no benefit for AIDS sufferers, and the MCC refused permission for formal clinical trials of the drug even after undergoing a politically-motivated restructuring to make it more sympathetic to Minister Zuma [107, 123, 125]. The Virodene saga paved the way for a much larger HIV-scandal – AIDS denialism – which would continue well into the next decade and would cost South Africa hundreds of thousands of lives [123, 126, 127].

### 1999 to 2006: AIDS denialism erodes public trust and efforts to contain private sector costs have limited success

By the end of the GNU era, the opportunity to move forward with the HSR agenda seemed to have passed, and in the 1999 to 2006 period, the fight to compel the state to roll-out a HIV treatment programme garnered far more policy-maker and civil-society attention than the HSR agenda. In addition, while some efforts were made in this period to regulate the private health sector to control costs (and set the stage for HSR), successes were limited.

At the start of the new millennium, 20% of pregnant women and nearly half of the armed forces were HIV-positive, and AIDS was the leading cause of death in the country [111, 125]. In this context, the Vissers introduced Mbeki (soon to be South Africa’s president) to a newspaper article by a magistrate with no training in medical science which drew on AIDS denialist rhetoric from the USA, and argued in defence of Minister Zuma, who was resisting the introduction of antiretrovirals (ARVs) for the prevention of mother-to-child transmission (PMTCT) of HIV [123]. Mbeki responded by attacking AIDS researchers, and delaying the introduction of ARVs in South Africa [123]. After being appointed Mandela’s successor, Mbeki, along with new Minister of Health Manto Tshabalala-Msimang, questioned the connection between HIV and AIDS, the accuracy of AIDS tests, and the safety and efficacy of AIDS treatments, arguing instead that AIDS was a Western conspiracy based on racist stereotypes of African sexuality [126, 128]. As a result of the government’s failure to respond to the epidemic, by 2005 life expectancy in South Africa had fallen to 48, down from 64 in 1994 [64].

The AIDS crisis and the ANC government’s response thereto significantly undermined public trust in the state’s ability to steward a public sector increasingly under strain, and, for some, exposed the Party’s commitment to establishing a universal, equitable, PHC-focused health system as purely rhetorical [59, 107]. However, AIDS and AIDS denialism also set the scene for civil society action, led by the Treatment Action Campaign (TAC), that would offer a counter argument to neoliberal forms of governance in two areas: The limits of the state’s responsibility to provide health care, and the obligation of the state to regulate markets for the public good.

The TAC was established in late-1998, initially to fight for a PMTCT programme that would ensure HIV-positive mothers had access to ARVs [129]. In 1999 Minister Zuma claimed that budget shortfalls prevented the Department from rolling out ARVs to HIV-positive women [111, 130]. At the time, the branded Azidothymidine cost $240 per month in South Africa, and it was generally assumed that AIDS drugs were simply too expensive for widespread use in developing countries (even though a generic version produced in India cost less than $50 per month) [111, 131, 132]. Generic substitution in the public and private sectors was a key tenet of the ANC’s HSR policy proposal in 1994, and the 1997 Medicines Act, passed under Minister Zuma’s leadership, made provisions for parallel imports and compulsory licensing (allowing the state to grant rights to local manufacturers to make generic versions of drugs without the permission of the patent-holder) [110, 111]. While both these provisions were allowed under the Agreement on Trade-Related Aspects of Intellectual Property Rights (TRIPS agreement) in health emergencies, the implementation of the Act was blocked for 3 years by a challenge in the Constitutional Court brought against the South African government by a collection of multinational drug companies under the umbrella of the Pharmaceutical Manufacturer’s Association (PMA) [7, 111, 126, 131]. Alongside the legal challenge, United States officials and representatives (including vice-president Al Gore – whose presidential bid the following year was funded by pharmaceutical companies) launched a massive lobbying effort to pressure Minister Dlamini-Zuma to revoke the offending clause of the Medicines Act [111]. In 2001, the TAC was admitted by the Pretoria High Court as a ‘friend of the court’ on the case [129]. The TAC argued that health is a constitutionally-enshrined human right that the state has a legal duty to protect, that excessive pricing of essential medicines by pharmaceutical companies violated these rights, and that the intellectual property rights imbued by the 1995 TRIPS agreement was not an inherent human right and therefore limitations on these rights were justified [129, 133]. Eventually, massive public demonstrations and global advocacy efforts by international non-governmental organisations (NGOs) such as Médecins Sans Frontières and Oxfam garnered enough public pressure to compel the PMA to withdraw the case [129, 131–133].

However, the TAC’s victory against the PMA occurred in the context of AIDS denialism – another hurdle to be overcome before South Africans would be guaranteed access to ARVs. In 2000, the findings of a local clinical trial supported the use of Nevirapine for PMTCT [130]. However, Minister Tshabalala-Msimang stalled, saying that even once Nevirapine was registered for PMTCT by the MCC, the provision of Nevirapine must first be tested for ‘feasibility’ at two pilot sites in each province (due to begin in March 2001), and even then implementation would be ‘phased’ [130]. In addition, there were reports that the MCC’s registration of the drug was deliberately delayed by some employees kowtowing to government [130]. Eventually, the PMTCT programme was initiated but restricted to ‘research and training’ sites – leaving most in need of treatment without access [66, 130].

The TAC, together with a coalition of paediatricians and children’s rights advocates, lodged a court case against Minister Tshabalala-Msimang for failing to implement a PMTCT programme that would ensure broad-based access to Nevirapine [66, 122, 130]. Amidst a TAC-led public mobilisation campaign that attracted much public support and media attention, a judgement was handed down in December 2001 in favour of the TAC’s application, concluding that a “countrywide [P]MTCT programme is an ineluctable obligation of the state” [66, 130]. The Minister appealed the decision, and claimed the Department could not take a decision on whether to scale-up the roll-out of Nevirapine until the pilot sites had been running for a full year (despite an evaluation by the Health Systems Trust already recommending the expansion of PMCTC in all provinces), but a final judgement handed down by the Constitutional Court in July 2002 once again found in favour of the TAC [130, 132]. The TAC’s successful fight to lower the price of AIDS drugs and compel the state to provide treatment for HIV arguably served to reaffirm the responsibilities of the state with regard to the regulation of the private sector and the provision of health services [129].

The HSR process stalled somewhat during the AIDS denialism era [7]. The 2002 Committee of Inquiry into Comprehensive Social Security in South Africa, or Taylor Committee, explicitly called for a unified NHS in which the public sector would remain the ‘backbone’ of the health system, but only as a long-term strategy [134, 135]. In the short-term, the Taylor Committee recommended a particularly progressive SHI [136]. In 2005, a Ministerial Task Team established to decide which of the Taylor Committee proposals to take forward, concluded that NHI was not feasible, and suggested pursuing SHI. However, there were concerns about the feasibility of a risk equalisation fund (REF, which requires substantial technical capacity) and the challenges of adequate risk pooling in a multi-purchaser environment [7, 136]. As a result these recommendations were largely ignored until the idea began to re-emerge in 2007 [7, 136].

While the AIDS crisis distracted public and policy-maker attention from the HSR agenda, it occurred alongside, and arguably exacerbated, growing health sector challenges caused by the public-private divide [7, 33]. As stated above, despite growing inequities between public and private sectors – by the end of the decade 73% of all doctors worked in the private sector, and by the mid-2000s the value of the tax subsidy per medical scheme member exceeded government expenditure on health per public sector beneficiary and amounted to 20% of the entire public health budget [74, 78] – in the early years of the new democracy no significant moves were made to regulate the private sector more tightly. However, in the late 1990s, regulatory efforts were made with respect to both private providers and private funders.

With respect to private providers, the 1997 White Paper for the Transformation of the Health System in South Africa suggested the development of a unified NHS in which public and private resources would be pooled and private health practitioners would be integrated with the public sector [137]. The White Paper also spoke of contractual arrangements and tariff negotiations to facilitate the utilisation of private sector beds by public sector patients, and introduced the idea of a certificate of need – which would ensure equitable geographic distribution of health facilities by limiting the licensing of new private facilities in areas with sufficient coverage [137]. The idea of a certificate of need was raised again in the 1999 Health Sector Strategic Framework and the 2003 National Health Act, but was never implemented, and was eventually successfully challenged in the Constitutional Court in 2015 by the Hospital Association of South Africa and the South African Dental Association [7, 138, 139]. Little was done to tackle concentration of ownership in the private sector, and by 2007 84% of private hospital beds were owned by three hospital groups [134].

In addition, efforts to contain costs in the private sector proved unsuccessful. In 2003, complaints were registered with the Competition Commission against SAMA and BHF for the publication of industry-wide tariffs that set ostensibly fair rates for various medical services and that medical schemes used to guide reimbursement rates [7, 140]. The Commission concluded that the tariff schedule was anti-competitive and demanded that SAMA and BHF cease the practice [7, 140]. However, by applying market logic in an industry where payments are made by a third party (medical schemes), this ruling further exacerbated high costs in the private sector. The 2004 National Health Act gave the Council for Medical Schemes (the regulatory body for medical schemes) the authority to establish an NHRPL to standardise what providers charge and funders reimburse [140]. However, the NHRPL was non-binding and did not successfully contain costs – while medical schemes used the NHRPL to determine reimbursement rates, providers continued to charge rates in excess of the tariff, resulting in members paying the remaining amount out-of-pocket [140–142].

More was done to regulate the private financing sector, beginning in 1998 with the MSA [143]. The Act, which was implemented in 2000, regulated medical schemes in favour of beneficiaries in order to expand the group of people with medical scheme coverage, and although not explicitly stated in the Act, was understood to be an attempt to lay the foundation for SHI [112, 136]. Under the MSA, medical schemes are not permitted to risk-rate or exclude individuals on the basis of age or health status, and are prohibited from applying repayment limits and ‘dumping’ patients on the public sector when their benefits run out [106, 144]. However, the MSA was intended to be implemented in concert with the White Paper, and so while ensuring *de re* access to medical schemes, in the absence of a system of tariffs, these regulations did little to contain costs [106, 137].

As such, costs in the private sector continued to rise due to a combination of over-utilisation, over-servicing, and high administration fees,^14^ and medical scheme membership contributions were increasingly unaffordable even for relatively well-off South Africans [74, 134]. Despite the MSA, medical scheme membership did not rise, and in 2006 medical scheme coverage was at its lowest point since 2002 [134, 145]. Rising private sector costs also meant increasing amounts of public funds being spent in the private health sector, for example, because the state subsidises the medical scheme contributions of civil servants, and through tax subsidies on medical scheme contributions [65, 66, 74].

### 2007 to 2015: Zuma reignites the health system reform agenda, but corruption and governance failures further undermine public trust

In this context of an increasingly unaffordable and unsustainable private sector, and growing doubt and impatience about the ability of neoliberal macro-economic strategies to fulfil the promises of the new South Africa, the ascendence of Jacob Zuma to the presidency both brought renewed hope for the HSR agenda, and introduced a grand-scale corruption scandal that arguably presented the final nail in the coffin of public trust in the state.

Jacob Zuma was deputy President in Mbeki’s government, but was dismissed by Mbeki in 2005 after courts found that Zuma was involved in a ‘generally corrupt relationship’ with infamous businessman Schabir Shaik [100, 146]. Although he was not formally charged with corruption at the time, in 2007, shortly after decisively winning the Presidency of the ANC from Mbeki, Zuma was served with papers by the Scorpions (a dedicated anti-corruption unit set up by Mbeki) to stand trial in the High Court on corruption charges [146–148]. While there is no doubt that Zuma was corrupt, it is also true that corruption was already flourishing in the South African government under both Mbeki and Mandela, and that Mandela in fact inherited a corrupt civil service from the apartheid government [31, 147, 148].

The political saga between Mbeki and Zuma was framed in the media as reflective of larger ideological tension between rational, economically judicious leadership and the rule of law on the one hand, and populism and corruption on the other [147].^15^, ^16^Zuma was a populist president in that he encompassed authoritarian, anti-intellectual, militaristic, cultural and patriarchal respectability, and pro-poor ideas [128, 132, 147, 148]. In addition, Zuma’s rise to the presidency took place in a period in which popular discontent at the consequences of neoliberal policies was growing [132], and his presidential campaign took advantage of general disaffection with Mbeki’s prioritisation of growth over redistribution [136]. In the Zuma-Mbeki divide, Zuma also had the support of the SACP and COSATU – both important to the ANC’s political legitimacy and election prospects [7]. Zuma’s political identity stood in sharp contrast with a vision of Mbeki as modern, intellectual, rational, unfeeling, autocratic and neoliberal [128, 147, 148]. Further, Mbeki can be understood as anti-revolutionary in that he sought not only to neutralise the revolutionary potential of the masses, but also to prioritise neoliberalism and an alliance with corporate capital [147, 148].

Thus, Zuma’s presidency renewed hopes for a more developmental state and paved the way for HSR in the form of a NHI as part of a populist agenda [33, 136, 148]. In 2007, at the ANC annual conference in Polokwane (where Zuma was elected as ANC president), the urgent implementation of the NHI was adopted as official ANC policy, and an ANC NHI Task Team (led by long-time NHI proponent Olive Shisana) was appointed to further develop the policy [65, 136, 153]. COSATU lobbied strongly for the NHI at the conference, and would remain a strong supporter of a single-purchaser NHI throughout the Zuma presidency [7, 65]. At the same conference, the ANC committed to a more radical approach to land reform which would see the market-driven, ‘willing-seller, willing-buyer’ approach (which had hereto prevented the government from reaching its redistributive goals) discarded [154, 155].

The global context of the time may also have contributed to putting HSR back on the policy agenda. The 2008 global financial crisis “loosened neoliberalism’s hold on policy” [156]. In an article entitled ‘Beware brain washing’ criticising the hegemony of neoliberal ideas in higher education, then Minister of Higher education, Blade Nzimande, warned that any policy proposal that challenged the “neoliberal agenda and elite class interests” would be met with resistance. In addition, in 2008 and 2009 United States President Barack Obama’s plans for health care reform in that country was being widely reported in South African media.^17^ Also at around this time, in 2010 the WHO’s World Health Report identified UHC, including purchaser-provider split and inclusion of the private sector in provision, as a guiding principle, and the idea quickly gathered momentum [4, 7].

However, NHI was still a contentious issue. A document containing the ANC NHI Task Team proposal – including the establishment of an NHI authority to take over funding and purchasing of the health care for the entire population and scrapping the medical scheme tax deduction – was leaked to the press, sparking backlash over the ‘demise’ of medical schemes, the cost of the NHI, and the ‘undue’ burden it would place on the country’s tax-payers [65, 162, 163]. In 2008 both the National Health Amendment Bill (seeking to provide for the appointment of a regulator for health pricing, and for a framework for collective and individual bargaining for health prices) and the Medical Schemes Amendment Act (seeking to establish a REF, and to make provisions for Low-Income Medical Schemes) – failed to pass through parliament [27, 140, 164].

In 2009, the ANC’s election manifesto promised the introduction of an NHI that would be publicly funded, publicly administered, free at the point of service, and would engage with private hospitals and group practices to encourage them to participate in the NHI [165]. After taking office, Zuma used his first State of the Nation address to commit to the implementation of the NHI [136]. Shortly thereafter, an NHI Ministerial Advisory Committee was established to advise the minster on policy and legislation for the NHI [65, 165]. Like the ANC NHI Task Team, the Ministerial Advisory Committee was chaired by Shisana, enabling the cross-pollination of ideas between the two [65].

Zuma also introduced Aaron Motsoaledi as the new Minister of Health, who unlike Tshabalala-Msimang, proved to be a passionate advocate for the NHI [7, 33]. Motsoaledi’s argument for the NHI centred on inequalities between the public and private sectors, and framed the NHI as an opportunity to pool the public and private sectors to eliminate these inequities and create a more efficient health system [27, 65].

By 2007, the number of private hospital beds per medical scheme beneficiary was twice that available to those dependent on the public sector, a general practitioner (GP) in the public sector served between seven and 17 times as many patients as a GP in the private sector, and there was a 23-fold difference in the number of specialist doctors per beneficiary between the public and private sectors [134]. Accordingly, whereas the pre-2008 version of the NHI focused on expanding medical scheme coverage and introducing mechanisms for cross-subsidisation between pools, post-2008 the policy proposals cemented around a single-payer system with a single funding pool to ensure equity and social solidarity [27, 113, 166].

Under Motsoaledi’s leadership there was a flurry of policy development activity, demonstrating the technical, economic and political challenges of HSR. In August 2011, after an intensely political policy development process, the NHI Green Paper was released [27]. The Green Paper proposed a tax increase of 3% to fund the NHI, a reduction from the 5% previously proposed, reflecting concerns about the burden on tax payers [113, 167]. Even so, in 2012, Minister of Finance Pravin Gordhan noted concerns about funding shortfalls for the NHI and said that additional funding options (including VAT increases, an employer’s payroll tax, a surcharge on the taxable income of individuals, or the introduction of user fees) had to be considered [168]. In addition, the Green Paper extended the implementation timeline for the NHI from 5 years to 15 years, with the first 5 reserved for reforming the public health system [167], while at the ANC’s 2012 annual conference it was resolved that the NHI must be set up using state revenue by 2014.

The Green paper also committed to a programme of PHC re-engineering to lay the foundation for a national health system centred on primary and preventive care, and to an NHI piloting process [113]. PHC re-engineering would involve the establishment of municipal ward-based PHC outreach teams, district-based clinical specialist teams focusing on maternal and child health, and school-based provision of PHC services [113, 169, 170]. Later, PHC re-engineering would be understood to include using district-level contracting units for PHC (CUPs) to contract multi-disciplinary networks of non-specialist private providers to provide PHC [7, 171, 172].

The NHI piloting – to begin in March 2012 – would be achieved through the establishment of 10 NHI pilot sites to resolve technical complexities and explore options for managing the public-private split, such as how best to contract and reimburse private providers [33, 65, 113]. When the pilot sites were established in 2012, Motsoaledi put the development of the White Paper on hold to allow time to learn from the piloting [33, 65]. The pilot sites revealed the challenges involved in contracting private providers to work for the public sector. Firstly, central government did not have the mandate for hiring and firing – this fell to Provincial governments which were not invested in the success of the pilot sites (particularly given that the NHI will likely see a reduction in the powers of provincial governments) [65, 173]. Secondly, very few doctors were willing to work in the pilot sites, perhaps because the reimbursement rates were too low [65, 173].

In 2014, an election year, Zuma announced the launch of Operation Phakisa (‘hurry up’). Like PHC re-engineering, Operation Phakisa was considered an important step towards improving the public sector in preparation for NHI – which would use data collected from a set of 10 Ideal Clinic learning sites established in 2013 to devise a set of initiatives that every clinic could use to improve service delivery [7, 65, 171]. Simultaneously, an inquiry into the South African Health Market (or the Health Market Inquiry – HMI) was initiated by the Competition Commission to examine the causes of high costs in the private sector – another significant challenge to implementation of the NHI [65].

Then, in 2015, a draft White Paper – National Health Insurance for South African: Towards Universal Health Coverage – was released for comment, describing a single-payer system in which a central authority contract with public and private GPs and specialists, and in which medical schemes would be reduced to providing ‘complementary’ coverage only [7, 27, 174]. The White Paper was labelled ‘Version 40’ – an indication of the many iterations developed since 2011, and of the contentious nature of the issue [7]. In particular, during this intervening period, Treasury continued to argue for a multi-payer system combined with a ‘solidarity tax’, on the grounds that it would allow medical scheme members to keep their coverage [65, 175]. Motsoaledi himself said that the various iterations were due to his desire to respond to technical criticism from experts and academics, while ensuring that the White Paper would be understandable for ‘every South African’ [65].

Alongside this contentious policy development process, the country was suffering a series of corruption scandals and governance failures that significantly undermined public trust.^18^ Perhaps the defining corruption scandal of the new democracy was the 1999 Arms Deal, under Mbeki’s presidency,^19^ in which the ‘Strategic Defence Package’ to modernise South Africa’s defence capacities saw contracts totalling tens of billions of rands improperly awarded to various arms manufacturers in exchange for bribes paid (reportedly) to Mbeki and Zuma, among others [100, 148, 178]. At the time, there was controversy over whether spending on defence was appropriate or necessary given the need for spending on social services [178–180]. The scandal received significant media coverage that was reignited in the mid-2000s [178, 181]. In May 2005, Shabir Shaik, long-time associate of Zuma, was convicted of having paid bribes to Zuma (then member of the Executive Council of the Economic Affairs and Tourism in KwaZulu-Natal province) in connection to the Arms Deal [146, 176, 178]. When Zuma himself was charged in 2007, he was able to build a network of supporters within, and exert political pressure on, the National Prosecuting Authority to protect him from prosecution for his involvement in the Arms Deal, and subsequent corruption cases [146, 148, 178]. Shortly before the 2009 general election, after Mbeki was recalled by the ANC and resigned the presidency, the National Prosecuting Authority dropped all charges against Zuma [128, 146]. After the ANC won the 2009 general election, and Zuma was installed as president of the country, he promptly disbanded the Scorpions [146].

From then on, Zuma’s presidency was characterised by ongoing corruption and governance failures that drew significant public attention. In 2008, the country suffered its first round of load-shedding – wide-spread, planned power outages necessary to preserve the country’s electrical power as a consequence of the operational and governance crisis in electrical parastatal, Eskom [182]. Load-shedding is causally connected both to neoliberal privatisation and corporatisation policies (with investment in generation capacity having been undermined by attempts to unbundle Eskom and facilitate the entry of private generators^20^) and corruption (with Zuma having facilitated and benefitted from the appointment of corrupt board members in 2011) [148, 176, 182, 183]. In August 2012, a miners strike in Marikana, North West Province (a mine owned by global company, Lonmin) descended into deadly violence, wherein 34 striking miners were gunned down by police [132, 147, 184]. Some of the violence was broadcast live by national and international media, and the brutality of the events, combined with the stark similarity of the images to the many instances of mass police brutality under apartheid, garnered significant public attention [184]. Zuma subsequently decided on the terms of reference of the commission established to investigate responsibility for the massacre and ensured that government institutions would not be held accountable, [184, 185]. In 2009, Zuma began security upgrades to his private residence at Nkandla, KwaZulu-Natal, authorised by the Minister of Public Works, and media articles reported on the ‘opulent’ and ‘excessive’ expenditure, leading to one of the most controversial and illustrative corruption cases in the ‘new’ South Africa [176, 186]. The Public Protector launched an investigation in 2013, finding maladministration, and recommending that Zuma pay back a reasonable percentage of the costs of the upgrades [176, 186]. Following a 2015 Constitutional Court case brought by an opposition party finding that the recommendations of the Public Protector are binding, Zuma paid back R7.8 million by securing a loan from VBS bank, with the loan itself revealed as a product of corruption, and the bank being placed under curatorship shortly thereafter [176, 178].

By October 2016, the scale of Zuma’s corruption was clear and the idea of ‘state capture’^21^ was firmly cemented in the public consciousness and understood as a national crisis [148, 176]. Zuma’s ‘weekend cabinet reshuffle’ in March 2017, understood as a final push in his attempt to co-opt the state for private gain, prompted a wave of civil society mobilisation, protest action, another motion of no confidence, and Zuma being recalled by the ANC as president of South Africa [148, 187, 188]. Zuma finally resigned the presidency in February 2018, and was succeeded by Cyril Ramaphosa [148, 189].

These events, alongside other major Zuma-linked scandals severely undermined public trust not only in Zuma, but also in the state [103, 176, 190]. After a brief moment of optimism in 2004-5, which some argue represent a pinnacle of trust in government, Zuma’s presidential term saw a steady decline in trust in national, provincial and local government,^22^ and in the police [132, 189, 190].

### 2016 to 2019: Zuma’s shadow continues to plague health system reform efforts amid doubt about the state’s capacity to regulate the private sector and deliver adequate public healthcare

While the NHI remained a key policy priority for the ANC, and Motsoaledi continued to drive the process forward, the loss of public trust and the fault-lines within government caused by state capture would intersect in various ways with the NHI policy process. After the release of the NHI White Paper in 2015, it was necessary to develop the details for financing and implementing the NHI. In 2017, an amended version of the NHI White Paper was released, alongside a policy paper on NHI implementation [171, 191]. The release of the Davis Tax Committee (an advisory committee established in 2013 to explore how government could raise revenue to meet its policy objectives, including NHI) report in 2017 raised concerns about financing of the NHI, saying that in the absence of sustained economic growth, the NHI is not financially sustainable in South Africa [192]. Despite this, and despite the fact that the detailed funding options for NHI promised by Treasury were not forthcoming, in June 2018 the NHI Draft Bill was gazetted and public comment invited by parliament [7, 193]. Along with the Draft NHI Bill, a Draft Medical Schemes Amendment Bill and Draft National Quality Improvement Plan were released. The Medical Schemes Amendment Bill was intended, among other things, to make the necessary changes to the private financing sector in preparation for the implementation of the NHI [194–196].

 However, the policy process was significantly more complex in the shadow of state capture and Zuma’s ousting. Firstly, while the tumultuousness of the Zuma presidency weakened the ANC’s hold on the country’s electorate, with the result that NHI became all the more important to the Party’s electoral prospects [7], state-capture and large-scale corruption in parastatals such as Eskom were frequently used to justify resistance to NHI in public submissions on NHI policy documents. 23

Secondly, Motsoaledi’s legislative push was complicated both by conflicting views on the appropriate role of the private sector in the NHI, and (politically motivated) opposition from COSATU [7]. During the 2016 political crisis preceding Zuma’s ousting, COSATU accused Motsoaledi and Treasury of deliberately sabotaging the NHI by kowtowing to private interests and ‘handing over’ the NHI to ‘private interests’, and called on Zuma (who COSATU supported until the cabinet reshuffle in 2016) to stop Motsoaledi’s ‘attacks’ on the NHI [7, 33, 65]. At the same time, Zuma accused Minister of Finance Gordhan of being in the pocket of ‘white monopoly capital’ and tried to oust him through the cabinet reshuffle, while Motsoaledi was among those calling for Zuma to step down [7].

Motsoaledi continued to argue passionately for the NHI on the grounds that the public-private divide created a two-tier health system that necessarily resulted in inequities (see, for example [202]). However, these arguments were seen as antagonistic to the private sector and alienated many private sector users, resulting in considerable contestation [7]. In addition, Motsoaledi was labelled as ‘driven by ideology’, unable to understand the technical and structural issues at play, and unwilling to listen to experts [7]. After the release of the 2015 White Paper on NHI, Motsoaledi, apparently responding to concerns within the ANC that restricting access to medical schemes would alienate much-needed middle class voters, said that the White Paper gave the wrong impression by implying that under NHI people will not be able to opt-in to private medical scheme coverage [203].

The challenge of reaching popular consensus on the implementation of the NHI was further complicated by increasing doubt about the capacity of the state to deliver health services [29]. The Life Esidimeni tragedy was a high-profile governance failure within the health sector that exemplified the problem. In 2015, the Gauteng Provincial DoH, seeking to reduce costs, took the decision to terminate a long-standing contract with Life Healthcare Esidimeni private hospital for the in-patient care and rehabilitation of patients with chronic psychiatric disorders, opting to discharge some patients, refer some to NGOs for further care, or transfer patients to public hospitals with psychiatric wards [204, 205]. However, it soon became clear that, in addition to the transfer process being ‘chaotic’ and ‘inhumane’, many patients were transferred to NGOs without the appropriate capacity to care for them [206–209]. The Health Ombud found in 2017 that a total of 91 patients died, and that all 27 of the NGOs to which patients were transferred were operating without valid licenses [209]. The Life Esidimeni saga therefore reinforced existing public perceptions of poor quality care in the public sector [210, 211].

Major scandals aside, in general service delivery in the public health sector was described as characterised by leadership and governance failures, inept, unprofessional and uncaring behaviour on the part of healthcare workers and poor quality of care, and a 2018 Office of Health Standards Compliance report found that on average, the facilities inspected met less than 50% of the required quality standards [7, 212, 213]. Corruption is also widely reported in provincial health departments – the site of the majority of public spending on health [29]. A 2016 analysis based on auditor general reports found that by 2013 6.3% of provincial health expenditure was classified as ‘irregular’ [29]. Corruption in provincial health departments is also regularly reported in the media [29]. By 2019, the idea that the public health system had ‘collapsed’ was frequently touted in the media [33].

Whether the result of pernicious ‘behind-the-scenes’ lobbying by private actors, the need to placate middle class voters and those concerned that the publicly delivered health services are inadequate, or a pragmatic recognition that the economic and political realities in South Africa mean the only possible NHI is one that incorporates private actors, over the course of his tenure Motsoaledi was increasingly open to engaging with the private sector in developing the NHI [7].

However, in parallel to these developments, the HMI was wrapping up, and its findings would reveal the extent of the challenges facing the private health sector. A provisional report was released in 2018, soon after the publication of the draft NHI Bill, and a final report released in 2019 [177, 211, 214]. The reports revealed a lack of regulation of the private sector and limited accountability of private providers resulting in over-servicing, supply-induced demand and rising costs [177, 211]. These challenges were reflected in the lived experiences of private sector users. While overall health financing in South Africa was progressive, among medical scheme members, poorer groups were paying a larger percentage of their income than richer groups, making contributions increasingly unaffordable for many [215]. In addition, medical scheme benefit packages were increasingly restrictive (meaning more and more services and products are not covered), and co-payments were common, with the result that many medical scheme members faced significant out-of-pocket expenditure [211, 215]. The final HMI report largely placed the blame for this state of affairs on the state’s failure to regulate the private sector appropriately, and recommended the institution of a supply-side regulator of healthcare to regulate hospitals and practitioners, an outcomes monitoring organisation to provide patients and funders with information on the health outcomes of providers and facilities, and a single standardised benefit option to be available across all medical schemes to enable consumers to compare prices and benefits across medical schemes more easily [177].

In July 2019, shortly after Motsoaledi’s tenure as Minister of Health came to an end in May, the National Health Insurance Bill was published [33, 216]. The Bill made provision for a single purchaser, single payer NHI, in which a National Health Insurance Fund (NHIF) would contract with ‘accredited health care service providers’ ‘in the public or private sector’ [216, 217]. Private medical schemes would only be allowed for services not covered by the NHI [216, 217]. The NHIF would be financed through general tax revenue, reallocation of medical scheme tax credit, employer and employee payroll tax, and an earmarked surcharge on personal income tax [216].

In this period, as had been the case throughout South Africa’s history, HSR efforts were significantly influenced by prevailing political concerns. While the HMI report revealed the harmful consequences of inadequate regulation of the private sector, the series of corruption scandals and governance failures in recent years, both in the health sector and more broadly, gave rise to concerns that that the state lacks the capacity to manage the NHI. Nonetheless, the process to finalise the NHI Bill and move towards achieving UHC through NHI was ongoing.

## Discussion

This synthesis of the history of HSR efforts in South Africa reveals the myriad of historical and contextual forces that shape UHC reform processes. In South Africa politics; the power of private sector; competing policy priorities; budgetary constraints; and ideas, values and ideologies have been particularly important in constraining, and sometimes spurring, HSR efforts. In this section, we explicate the effects of these factors to reveal how the history of HSR in the country has shaped the social and political meaning of UHC in this context.

### Social and political specificities shaping health system reform policy processes in South Africa

Firstly, in keeping with Gilson’s [33] suggestion that health financing reform is a political process above all, political considerations have been a major determinant of what reforms are possible. For example, in the 1940s, the electoral victory of the National Party in 1948 and the institution of apartheid scuppered HSR plans and led to the dissolution of the health centre project [56]. In the late 1970s and 1980s, political pressure from (local and global) anti-apartheid opposition, alongside related budgetary pressures, informed the decision to deregulate and privatise the health sector [53]. The effects of this decision are complex. On the one hand, the resultant policy changes led to private-sector growth (including increased numbers of black South Africans using private providers and medical schemes), with the consequence that post-1994 HSR efforts faced resistance from a strong and well-established private sector [78]. On the other hand, concern that the state was abdicating its responsibility as a provider of health services and regulator of the private sector reignited interest in HSR among academics and civil society actors, and de-regulation facilitated a cost-explosion that would make the need for HSR all the more evident [31].

In the post-1994-era, while the AIDS crisis and AIDS denialism acted as a policy distraction and pushed HSR off the policy agenda, Zuma’s appointment as president successfully reignited the policy process for the NHI [33]. However, the political saga of the Zuma presidency, including state capture and Zuma’s eventual ousting, also increased opposition from COSATU to Motsoaledi’s softening stance on the involvement of the private sector [7].

The influence of the private sector, and popular ideas about the appropriate role of the private sector in the health system, have played a role in shaping what sorts of reforms are possible in South African since the 1920s. Both in the 1926–1939 period, and in the 1940s, MASA’s support for universal HSR helped to push the policy process forward [36]. However, in the late 1940s and 1950s MASA’s position changed – partly as a result of vested interests in the continuation of private practice for GPs, and partly because of an ideological reaction to the socialist overtones of Gluckman’s proposals – with the result that Gluckman was unable to make significant progress before the 1948 election of the National Party [36, 56]. Similarly, in the post-1994 era, the power of the private health sector has clearly shaped the nature of reforms. In the 1994–1997 period, technical experts deemed policy options that would exclude the private sector ‘unfeasible’ and gravitated toward policy options that would be more palatable to private sector actors and users [31]. More recently, over the course of his tenure (2009–2019) Motsoaledi was compelled to soften his stance on the role of the private sector in the NHI and increasingly engage private sector actors in the policy development process [7].

While it is not clear whether the increased involvement of the private sector in the NHI is a reflection of behind-the-scenes lobbying, beneficiary politics that prioritise private sector users, or pragmatic considerations of the relative capacities of the public and private sectors, it is clear that the scale of the private sector in South Africa has contributed to determining what sorts of reforms are feasible [7]. Furthermore, this analysis suggests that the concerns of private sector users have exerted more influence on the HSR process than those of the majority of South Africans, who rely predominantly on the public sector (and [33], see also [134]). This is evident, for example, in Treasury’s persistent opposition to NHI reform proposals that would negatively impact medical scheme members and wealthier taxpayers [7, 31]. Despite both the 1974 De Villiers Commission, and the HMI (launched in 2013 and concluded in 2019) demonstrating the pernicious influence of the private sector on the public sector, and the host of challenges faced by private sector users and medical scheme members, policy options that entail a scaling-back of the private sector continue to garner significant opposition.

The persistent power of the private sector is a function of past policy decisions that facilitated its rapid expansion in the 1970s and 1980s but is also enabled by the specific media culture in the country and, likely, by low levels of trust in the state. In South Africa, the media tends to positively represent the private sector, and to reflect the concerns and interests of private sector actors and users [7, 33]. Furthermore, public trust in government institutions generally has been declining since 1994 [190, 218, 219]. Corruption scandals and governance failures in health and other sectors, combined with evidence of declining quality of care in the public sector, cement the idea that state cannot be trusted to deliver health services or manage health funds, and that the private sector is a more appropriate mechanism for the delivery of healthcare. These socio-political specificities help to explain why the current policy proposal – for a single purchaser, single payer NHI in which services would be purchased on behalf of users from private or public providers [216, 217] - differs from the idea initially proposed by the ANC in the early 1990s for a NHS that would ensure universal access to publicly provided healthcare.

Furthermore, in the context of low public trust in the state, values-based arguments for HSR will likely prove insufficient to garnering public support. McIntyre and Van den Heever [134] point out that after the transition to democracy, there “was a considerable spirit of social solidarity and potentially a greater willingness to accept relatively large cross-subsidies,” and Motsoaledi frequently drew on solidarity as a value when speaking of the NHI. However, whether or not the ‘spirit of solidarity’ persists, public support for the NHI will remain low so long as there is broad popular doubt about the capacity of the state to manage reforms.

While a popular belief that the state lacks the capacity to, or cannot be trusted to, deliver NHI has made building popular support for HSR more difficult, it is also true that South Africa’s political history renders the inequities of the current system particularly problematic. South Africa’s history of racial segregation and oppression under colonialism and apartheid imbue the contemporary inequities in healthcare, and therefore the NHI, with a particular political meaning and social relevance. As McIntyre says, “given the political history of legislated discrimination on the basis of race under apartheid, there is clearly a desire to avoid health system differentials on the basis of class” [134]. In another context, UHC might have been achievable through a multi-purchaser model with tiered benefits. In South Africa, however, socio-economic inequities have a particular meaning that is connected to the country’s past. As a result, tiering is particularly problematic in this context.

In addition, while reducing financial risk as a result of paying for healthcare, is a central goal of UHC reforms in most contexts [4], South Africa’s UHC reforms are taking place in a context in which financial risk protection is already fairly robust [27]. The challenges that persist however, and which HSR efforts are intended to address include private sector costs, the quality of care in the public sector, and the inequities between the two sectors [134]. What UHC means in this context, therefore, is reforms that would ensure equity and solidarity, alongside financial risk protection. In the context of low trust in the state, however, this is difficult to achieve, and there is a risk that compromises made to increase popular and political support for reforms, such as the increased involvement of for-profit actors, could result in reforms that do not align with these social values [32].

### The value of the historical approach

Given this social and political complexity, analysing the health policy process longitudinally has allowed an understanding of the influence of actor positions, relational dynamics, and ideational factors, and exposed the historical tributaries of contemporary challenges. Firstly, it showed that declining trust in the state is likely contributing to popular opposition to HSR today. Further research on the influence of a legacy of corruption and governance failures, and the trust between the public and the state more broadly, on HSR processes is needed. With respect to moving forward with UHC reforms in South Africa, the historical analysis suggests that in addition to increasing popular support for reforms through values-based arguments that connect to the country’s particular history, policy-makers would do well to develop strategies to build trust in the state. Successful trust-building strategies would have to increase public trust in the state not only as a provider of health services (for example with well-publicised quality improvement projects) but also as a regulator and funder – both complex interventions.

Secondly the historical analyses helped to reveal the consistency of support or opposition by actors (see also [25]). In this case, for example, Treasury’s opposition to HSR proposals has remained relatively fixed over a long period of time – which might suggest that their position has as much to do with institutional culture or ideology as with the economic calculations at any particular moment. Similarly, the strength and longevity of the private sector, combined with low levels of trust in the state, reinforces the idea that the state is not an appropriate mechanisms for the delivery of health services, and increases opposition to reforms. The longitudinal perspective also highlights the historical tributaries of this state of affairs, including neoliberalism and policy decisions made under apartheid. With respect to opposition from Treasury, private sector actors and the general public, rhetorical strategies that recognise and respond to the ideational underpinnings of this opposition might prove fruitful in overcoming it.

## Conclusion

In South Africa, UHC is being used as a universal goal of health systems to justify and legitimate the establishment of an NHI, and for this reason South Africa may be counted among those countries undertaking health financing reforms in response to the global push for UHC. In reality, however, the NHI policy agenda predates the WHO’s endorsement of UHC as a global goal by over a decade, and the push for HSR has a nearly 100-year history in South Africa. Throughout this history of HSR efforts, a range of social and political realities, many of them unique to South Africa, have shaped the policy proposals and constrained the potential for change, and this long policy process, as well as South Africa’s unique political history, continue to influence the HSR efforts in important ways. In addition, the country’s political history has given rise to dominant ideas, values and ideologies that imbue HSR with a particular social meaning. While these ideas and values increase opposition and complicate reform efforts, they also help to expose the inequities of the current system and re-emphasise the need for reform.

These findings demonstrate that while UHC might be considered a universal goal of health systems, the process of implementing UHC reforms, and even the nature of those reforms, will be unique in every context – shaped by social, political and historical particularities. For analysts and reformers alike, this entails paying attention to the historical tributaries of contemporary challenges to reform, and recognising that the extent to which UHC reforms make a substantive difference to the lives of those most in need depends, at least in part, on the social meaning of UHC in any particular context.

## Data Availability

Data sharing is not applicable to this article as no datasets were generated or analysed during the current study. The materials used for this analysis are largely publicly available. Where not publicly available, the material is available from the corresponding author on reasonable request.

## Abbreviations

ANC: African National Congress
COSATU: Congress of South African Trade Unions
DoH: Department of Health
GNU: Government of National Unity
HCFC: Health Care Finance Committee
HMI: Health Market Inquiry
HSR: Health system reform
MASA: Medical Association of South Africa
MSA: Medical Schemes Act
NHI: National Health Insurance
NHRPL: National Health Reference Price List
NHS: National Health System
RDP: Reconstruction and Development Plan
REF: Risk Equalisation Fund
SACP: South African Communist Party
SHI: Social Health Insurance
UHC: Universal Health Coverage
WHO: World Health Organization

## Footnotes

1 See, for example, Gilson et al. 1999 [31], McIntyre et al. 2003 [32], van den Heever 2016 [27], Waterhouse et al. 2017 [7], Gilson 2019 [33].

2 Prior to 1910, from the 1652 arrival of the Dutch, South Africa was a Dutch and then a British colony [41].

3 Britain’s 1944 white Paper laying out the plans for the NHS on the basis of Beveridge’s recommendations states “everybody, irrespective of means, age, sex or occupation, shall have equal opportunity to benefit from the best and most up-to-date medical and allied services available” (quoted in WHO 2000 [54]).

4 This was in stark contrast to the health system at the time, which was hugely fragmented (each racial group had its own National Health Department, every homeland and provincial administration had a Department of Health, and each of the 400 local authorities had their own Health Departments), hospi-centric, and encouraged the flourishing of the private health sector [73].

5 It is important to note that while strongly influenced by Beveridge’s universalist ideas, Gluckman did not substantially challenge mainstream racist values, and the proposed health centres were still segregated [51].

6 In 1983 the Government budget allocation to the military was 18% of gross national product, compared to 3% to the health budget [43].

7 Until the 1970s, most private health providers had been mission hospitals and facilities owned by industry (such as mining companies) [78].

8 As part of the National Party’s grand Apartheid strategy (beginning in the 1950s) certain areas of South Africa were demarcated as ostensibly self-governing ‘bantustans’ or ‘homelands’ where black people could settle, justifying the non-enfranchisement of the black population in ‘white South Africa’ [71, 75, 86].

9 See for example Benatar 1985 [90], Coovadia et al. 1986 [88], Klopper 1986 [77], and Coovadia 1988 [89].

10 The guidelines eventually adopted at the 1992 ANC conference were less conciliatory and more radical, no longer spoke of ‘pragmatics over ideology’ and included state controls of financial institutions [95].

11 Human resource challenges were partly a consequence of the loss of international doctors at mission hospitals due to anti-apartheid boycotts, and of junior doctors fearing conscription under the apartheid government and fleeing political instability [73].

12 See, for example, Breier 1995 [114], Peacock 1995 [115], Staff reporter 1995b [117], Streek 1995 [116].

13 This is despite the fact that the Committee of Inquiry reported to the Director General of the DoH and not the Minister [31].

14 Medical schemes are not-for-profit entities but are administered by for-profit companies [31, 66]. Between 1992 and 1998, the cost of non-health expenditure (administration and brokerage) increased 243.5%, and by 2003 the cost of MS administration was 4.5 billion [66].

15 See for example Mde and Brown (2005) [149], Staff Reporter (2005) [150], Rantao (2005) [151] and Mgwaba (2005) [152].

16 While there is no doubt that Zuma was corrupt, it is also true that corruption was already flourishing in the South African government under both Mbeki and Mandela, and that Mandela in fact inherited a corrupt civil service from the apartheid government. In addition, some commentators have argued that given the neoliberal macro-economic strategy of the ANC, corruption and patronage were necessary in order to create a black capitalist class that could support the ANC politically and economically [31, 147, 148].

17 See, for example, Obama 2008 [157], Sidley 2008 [160], Pollitt 2009 [158], Vanden Heuvel 2009 [159] and Balz 2010 [161].

18 It is important to recognise that corruption in South Africa is by no means unique to the Zuma government, or the ANC government more broadly. There were many instances of corruption under the apartheid regime [76, 176], and fraud and corruption are also common in the private sector [177].

19 The ‘corrupt Zuma vs. anti-corrupt Mbeki’ narrative is questionable given that Mbeki both benefitted from, and quashed investigation into, the Arms Deal, but some commentators argue nonetheless that Mbeki’s ‘successful image management’ means that the narrative holds true in the popular imagination [146, 147].

20 These attempts were ultimately successfully resisted by trade unions [103].

21 ‘State capture’ refers to the “capture of the state apparatus by private interests seeking to utilize state powers or resources to their advantage…and is used [in South Africa] to describe mutually-beneficial corrupt relations between former-President Jacob Zuma and his political allies, and the Gupta family” [182].

22 Zuma’s networks of patronage are known to extend to provincial and local governments [148, 187].

23 See, for example, IRR 2018 [197], Sect. 27 et al. 2018 [194], Dullah Omar Institute 2019 [198], SAMA 2019 [199], [184], Medical Brief 2022 [200], Solanki et al. 2022 [201].
